# Perampanel in brain tumor‐related epilepsy: Observational pilot study

**DOI:** 10.1002/brb3.1612

**Published:** 2020-04-14

**Authors:** Marta Maschio, Alessia Zarabla, Andrea Maialetti, Diana Giannarelli, Tatiana Koudriavtseva, Veronica Villani, Silvana Zannino

**Affiliations:** ^1^ Regina Elena Institute for Hospitalization and Care Scientific IRCCS ‐ Center for Tumor‐Related Epilepsy ‐ UOSD Neuroncology Rome Italy; ^2^ Regina Elena Institute for Hospitalization and Care Scientific IRCCS ‐ Biostatistic Unit Rome Italy; ^3^ Regina Elena Institute for Hospitalization and Care Scientific IRCCS ‐ UOSD Neuroncology Rome Italy

**Keywords:** antiepileptic drugs, brain tumors, BTRE, epilepsy, molecular factors, perampanel, QoL, responder rate

## Abstract

**Objective:**

Possible loss of efficacy and potential interactions between antiepileptic drugs (AEDs) and chemotherapy could complicate the management of patients with brain tumor‐related epilepsy (BTRE) that may expose patients to an increased risk of adverse events. Perampanel (PER) is a highly selective, noncompetitive, alpha‐amino‐3‐hydroxy‐5‐methyl‐4‐isoxazole propionic acid (AMPA)‐type glutamate receptor antagonist. This study evaluates the effectiveness, QoL, cognition, and mood of PER in add‐on therapy in BTRE patients.

**Material and Methods:**

Observational pilot study on the effectiveness of PER as add‐on therapy in BTRE patients with uncontrolled seizures with a 6‐month follow‐up.

**Results:**

We recruited 26 BTRE patients. During the follow‐up, 16 underwent chemotherapy and 11 radiotherapy; 11 had disease progression. Five patients dropped out. Mean daily PER dosage was 6.6 mg in the 21 patients who completed the follow‐up and 6.4 mg in the ITT population. The mean number of seizures/month decreased from 10.8 ± 15.03 at baseline to 1.7 ± 4.34 in the 21 patients who reached the final follow‐up. Responder rate was 88.4%: Eight patients were seizure‐free, 15 had ≥50% seizure reduction, and 3 remained stable. Four patients (15.4%) reported AEs: 2 required PER dose reduction, and 2 dropped out. Neuropsychological, mood, and QoL questionnaires were not statistically different compared to baseline. There were no significant differences in seizure control in patients with/without IDH1 mutation and with/without MGMT methylation.

**Conclusions:**

Perampanel proved to be effective on seizure control in BRTE patients and to be well tolerated without negative effects on cognition and QoL. Perampanel could be a valid therapeutic option in BTRE.

## Abstract 2

Epilepsy is a very common symptom of brain tumors, and AEDs should be selected especially by tolerability, and drug‐interaction potential. Observational pilot study on 26 brain tumor‐related epilepsy patients treated with PER in add‐on, with 33.3% seizure‐free after 6 months. Perampanel treatment brought a reduction in seizure frequency also in the group of patients with progression disease and seems well tolerated. No influence observed on PER efficacy by factors related to brain tumors, such as systemic therapy, progression, histology, or surgery. We obtained stability on patients' perceived QoL and cognitive domains and a low rate of neuropsychiatric adverse events.

## 1 INTRODUCTION

In patients with brain tumor, epilepsy represents one of the most important symptoms. It has been estimated that seizures occur in rates varying from 20% to 40% of patients with brain tumors; seizures are the onset symptoms in a significant proportion of cases (Maschio et al., 2014; Perucca, 2013; Van Breemen, Wilms, & Vecht, 2007).

Approximately 10% of patients with brain tumor do not have seizures as first symptom, but develop seizures at a later stage (Maschio, 2012).

Up to 90% of patients with glioneuronal tumors, diffuse low‐grade gliomas, grade II astrocytomas, oligodendrogliomas, and meningiomas develop pharmacoresistant epilepsy (Rudà, Bello, Duffau, & Soffietti, 2012). Additionally, brain tumor‐related epilepsy (BTRE) patients may have an increase of adverse event due to a possible interaction with antiepileptic drugs (AEDs) and anticancer therapies (Perucca, 2013).

Therefore, the selection of the appropriate AED therapy in BTRE patients should be driven by multiple factors, which include not only efficacy in the specific type of seizure to be treated, but also tolerability and drug‐interaction potential.

Between new AEDs, perampanel (PER) is the first‐in‐class, highly selective, noncompetitive, alpha‐amino‐3‐hydroxy‐5‐methyl‐4‐isoxazole propionic acid (AMPA)‐type glutamate receptor antagonist (Rogawski & Hanada, 2013).

Perampanel is approved as add‐on therapy for partial‐onset seizures with or without secondary generalization in adult patients with epilepsy (Raedler, 2016).

Perampanel pharmacokinetic profile is characterized by good bioavailability, rapid absorption, and a long elimination half‐life. Perampanel is metabolized mainly by CYP3A4 and CYP3A5 enzymes. The most common side effects that often disappear with dose reduction are as follows: dizziness, fatigue, psychomotor impairment, somnolence, vertigo, aggressiveness, mood disorders, and cognitive deficits (Meador et al., 2016; Rohracher et al., 2016; Snoeijen‐Schouwenaars, Ool, Tan, Schelhaas, & Majoie, 2017; Steinhoff et al., 2013; Trinka, Steinhoff, & Nikanorova, 2016; van Ool et al., 2016).

Three randomized, double‐blind, placebo‐controlled trials (French et al., 2012, 2013; Krauss et al., 2012) and their extension study (Krauss et al., 2014), proved that, in patients with focal seizures with/without secondary generalization, PER was efficacious and well tolerated, and the proportion of patients that remained on PER treatment was comparable to that of placebo.

For these reasons, we decided to study PER as add‐on therapy in BTRE patients to evaluate the efficacy on seizure control, as well as tolerability and impact on QoL, cognition and mood for 6 months (European Medicine Agency, 2018).

## 2 MATERIALS AND METHODS

Observational pilot cohort study was approved by the Ethical Committee (Prot. n° 0004691.12_04_2017). The study conforms to recognized standards of European Medicines Agency Guidelines for Good Clinical Practices.

### 2.1 Primary aim

To evaluate efficacy of PER as add‐on therapy on seizure control in BTRE patients, after 6 months.

### 2.2 Secondary aims

1. To obtain 50% of patients responders.
2. To detect the presence of PER‐related side effect during follow‐up period compared with baseline.
3. To monitor PER impact on cognition, mood, and Quality of life after 6 months of therapy, compared with baseline.

### 2.3 Population

BTRE patients with uncontrolled seizure activity despite AED therapy with adequate dosages. Perampanel was added as first or second add‐on. Patients were monitored for 6 months and underwent functional and neurological evaluation, count of seizure frequency, monitoring of adverse events, administration of QoL, mood, and cognitive questionnaires. All patients were treated with current standard care for brain tumor, including neuroradiological follow‐up. Any patient that experienced uncontrolled seizures with PER at maximum tolerated doses and needed a new AED was considered treatment failure and analyzed as such (Last Observation Carried Forward “LOCF” will give seizure frequency at AED change).

### 2.4 Investigational product

Perampanel as first or second add‐on therapy at dosage ranging from 4 to 12 mg/die was taken as a single oral dose at bedtime. The starting dosage is 2 mg/die with a slow increasing schedule as per label (with a weekly increase of 2 mg/day). Minimal effective dose is 4 mg/day. Depending on seizure control and on occurrence of adverse events, in order to achieve freedom from seizures, PER was titrated up to the maximum approved dosage of 12 mg/day.

### 2.5 Inclusion criteria

Patients age ≥ 18 years ≤ 75 (both sexes) with primary (low‐ and high‐grade WHO gliomas) or secondary brain tumor, with biopsy or surgical resection; in a stable phase of disease (evidenced by unchanged neuroradiological examinations), with/without chemotherapy (CT), radiotherapy (RT), and corticosteroids started before PER introduction. Structural epilepsy characterized by focal onset (aware/unaware seizures) (Scheffer et al., 2017); ≥4 seizures in the last month, despite 1–2 AEDs at the maximum tolerated stable dosages. Seizure count in the last month before the enrollment. All persons gave their informed consent prior to their inclusion in the study.

### 2.6 Exclusion criteria

Age ≤ 18 years ≥ 75 (both sexes) with primary BT; patients with cerebral lymphoma; patients with secondary BT (brain metastases); Karnofsky Performance Status (Karnofsky et al., 1951) < 60; Mini‐Mental State Examination (Folstein, Folstein, & McHugh, 1975) < 24; patients with other chronic neurological and psychiatric diseases; pregnancy or breastfeeding.

### 2.7 Study design

Trial duration for patient: 24 weeks + 1 week for titration of PER up to 4 mg/day.

Total trial duration including follow‐up: 49 weeks, 24 weeks for recruitment.

We decided a follow‐up period of 6 months, according to European Medicine Agency‐EMA guidelines (European Medicine Agency, 2018).

The study schedule is the following:

Visit 1 (Time 0): Inclusion and exclusion criteria check. Signature of informed consent. Complete neurological evaluation and check out of adverse events by Adverse Event Profile‐AEP (Gilliam et al., 2004) and patients' spontaneous report of adverse events classified according to National Cancer Institute‐Common Terminology Criteria for Adverse Events‐NCI‐CTCAE (Cancer Therapy Evaluation Program, 2003). Seizure count over the last 28 days.

Patients were given a seizure diary to compile in order to register any information about seizure occurrence in the last month. In order to attest seizure occurrence, patients were requested to call the center after each episode and calls were recorded and compared with questionnaire responses. Perampanel initial dosage of 2 mg/day. Patients underwent a neuropsychological battery of tests, including Quality of life and behavioral assessment.

Visit 2 (1 week): neurological evaluation, check of seizure frequency, adverse event monitoring using both AEP and patients' spontaneous report of adverse events. Perampanel up‐titration of 4 mg/day. Dose adjustment up to a maximum dose of 12 mg/day may be done along all treatment period miming clinical practice.
Visit 3 (3 MONTHS OF TREATMENT PERIOD): neurological examination, check of seizure frequency, adverse event monitoring using both AEP and patients' spontaneous report of adverse events, mood, and quality of life evaluation.
Visit 4 (6 MONTHS ASSESSMENT ‐ LAST STUDY VISIT): neurological examination, adverse event monitoring using both AEP and patients' spontaneous report of adverse events, neurocognitive, mood, and quality of life evaluation.

Seizure diary and Investigational Product check. Patients with improved seizure control continued PER therapy at the appropriate dosage with the marketed drug. If during PER treatment period at maximum tolerated dosage, patients reported seizure activity that requires the introduction of another AED, it would be considered as treatment failure and therefore analyzed as such (seizure frequency would be considered equal to that at study entry).

### 2.8 Primary efficacy variables

To evaluate efficacy of PER, we used the mean seizure frequency in comparison with baseline during the period of 6 months treatment, after having reached minimal effective dose of 4 mg/day and freedom from seizure rate at 6 months of treatment.

### 2.9 Secondary efficacy variables

Responders are defined as patients who obtained a ≥50% reduction of seizure frequency in comparison with baseline. Seizure frequency was evaluated as seizure frequency mean through the 6 months of treatment.

Perampanel‐related side effect were detected by mean values of AEP, and with spontaneous reports of adverse event at final follow‐up compared to baseline (visit 3 and 4). Perampanel impact on cognition, mood, and quality of life was assessed by mean values of neuropsychological tests, psychological state, and Quality of life questionnaires at final follow‐up compared to baseline (visit 3 and 4).

### 2.10 Safety variables

Incidence of adverse events using PER evaluated by AEP described later.

An “adverse event” (AE) is whichever unfavorable and unintended sign, symptom, or disease associated temporally with the use of a medical treatment or procedure that might or might not be considered related to the aforementioned treatment or procedure. Disease progression is not considered an AE. Patients who are administered at least one dose of drug will be included in the toxicity analysis. AEs (spontaneously reported or observed) will be recorded in association with details of time of onset and resolution, intensity, need for treatment, and possible connection with the ongoing treatment in the investigator's opinion.

### 2.11 Specific assessments/tools/scales

#### 2.11.1 Side effect evaluation

Presence of side effects was assessed using Adverse Events Profile (Gilliam et al., 2004): a self‐report multi‐item questionnaire specific for the evaluation of AEDs side effects in the last 4 weeks and with patients' spontaneous report of adverse events, classified according to National Cancer Institute‐Common Terminology Criteria for Adverse Events‐NCI‐CTCAE (Cancer Therapy Evaluation Program, 2003) a series of criteria to evaluate side effects in oncology.

#### 2.11.2 Quality of life and psychological state evaluation

Patients' perceived quality of life was evaluated by Quality of life in Epilepsy Inventory (QOLIE 31P‐v2) (Cramer & Van Hammée, 2003) a 31‐item self‐administered questionnaire for epileptic patients. Psychological state was assessed by Symptom Checklist‐90 (SCL‐90) (Derogatis & Savitz, 2000) a self‐administered 90‐item questionnaire, that assesses the intensity and frequency of psychopathological symptoms in the last week.

#### 2.11.3 Neurocognitive evaluation

We administered to patients a battery of standardized neuropsychological tests, which included the following: Mini‐Mental State Examination (MMSE) (Folstein et al., 1975)‐global cognitive status; Raven CPM (Basso, Capitani, & Laiacona, 1987)—nonverbal abstract reasoning; Trail Making Test (TMT) (Giovagnoli et al., 1996) and Visual Search (Spinnler & Tognoni, 1987)‐selective visual attention and oculo‐manual coordination; Rey auditory verbal learning test (RAVLT) (Carlesimo et al., 1996), immediate and delayed recall‐verbal learning; Rey–Osterrieth complex figure (ROCF) (Carlesimo, Buccione, & Fadda, 2002) immediate and delayed recall‐visuo‐spatial learning; and Rey–Osterrieth complex figure (ROCF) (Carlesimo et al., 2002) copy‐visuo‐spatial and visuo‐constructive abilities. Phonemic and semantic fluency (Novelli et al., 1986)—Verbal processing speed; Tower of London (ToL) (Krikorian, Bartok, & Gay, 1994)—executive functions.

### 2.12 Population size and statistical analysis

All enrolled patients will be considered as intention‐to‐treat population (ITT). Safety and efficacy of PER will be evaluated in this patients' population.

The primary endpoint will be the mean difference in the number of seizure pretreatment and after 6 months. We will use the *t* test for paired data.

Patients who do not reach 6 months of therapy will be evaluated in the last month of follow‐up available.

Based on an earlier study of a large population of drug‐resistant patients (Kanwaljit et al., 2016), we estimated an average seizure rate of 4 per month before the introduction of Perampanel; assuming that the treatment gives a reduction in the mean seizures number equal to 2 and estimating, from data of the preceding series, that this difference has a standard deviation (*SD*) of 2.8, 17 patients will be needed to obtain a statistical power of 80% to a level of significance of 5%.

## 3 RESULTS

We recruited 26 BTRE patients with structural epilepsy with focal seizures (16 males, mean age 47.5 years): 8 low‐grade gliomas, 8 high‐grade gliomas, 7 glioblastomas, 2 meningiomas, 1 metastasis. Eleven patients were on AEDs monotherapy (Phenobarbital‐PB: 1 patient; Lamotrigine‐LTG: 1 patient; Oxcarbazepine‐OXC: 1 patient; Zonisamide‐ZNS: 1 patient; Levetiracetam‐LEV: 7 patients) and 15 on polytherapy (see Table 1).

**TABLE 1:** Patients' clinical and vital data

Pat	Age (years)	Sex	Histology	Site of tumor	IDH1	MGMT	Surgery	CT	RT	Seizure type	No. of seizures in the month before entering the study	Baseline AED therapy	PER dose at final follow‐up (mg/day)	No. of seizures/month at final follow‐up	Drop out: months of follow‐up available and reasons	Seizure number/last F.U. available	Adverse events during PER therapy	Disease progression during PER follow‐up
1	75	M	GBM	Frontal	Mutated	Not methylated	GRT	Other‡	No	Focal aware seizure	5	LCM 300 LEV 3,000	6	1			No	Yes
2	48	M	GBM	Frontal	Not mutated	Methylated	GRT	Bevacizumab‡	No	Focal to bilateral tonic‐clonic	7	LCM 400 LEV 3,000	6	0			No	Yes
3	46	M	AOA	Multilobular	Not mutated	Methylated	Biopsy	Temozolomide‡	No	Focal to bilateral tonic‐clonic	30	VPA 1,500 LEV 3,000 LTG 200	6	0.5			No	No
4	40	F	LGA	Frontal	Unknown	Unknown	PR	No	No	Focal to bilateral tonic‐clonic	2	LTG 400	6	0.9			No	No
5	60	M	LGA	Temporal	Not mutated	Not methylated	PR	Temozolomide‡	No	Focal aware seizure	60	LCM 400 LEV 3,000	8	0			No	No
6	74	M	AA	Frontal	Not mutated	Methylated	PR	Temozolomide‡	No	Focal aware seizure	3	PB 100	8	0			No	No
7	42	M	LGA	Frontal	Unknown	Unknown	GRT	Fotemustine†	No	Focal unaware seizure	2	OXC 1,800	12	0			No	No
8	57	F	GBM	Multilobular	Unknown	Unknown	Biopsy	Fotemustine§	No	Focal aware seizure	30	LEV 3,000	8		2 (Death)	20	No	Yes
9	69	F	LGO	Parietal	Unknown	Methylated	PR	Temozolomide†	No	Focal aware seizure	24	LE 3,000‐LCM 400	6	0.8			No	No
10	34	M	AA	Frontal	Mutated	Methylated	GRT	Temozolomide‡	Yes†	Focal to bilateral tonic‐clonic	2	LEV 3,000	6	1			Vertigo (dose reduction)	Yes
11	36	M	LGA	Multilobular	Mutated	Unknown	Biopsy	Temozolomide	No	Focal unaware seizure	24	ZNS 300 OXC 1,500	8	1.8			No	Yes
12	37	M	AA	Temporal	Unknown	Unknown	GRT	Temozolomide†	No	Focal aware seizure	2	VPA 1,000 LEV 2,500	6	0			No	No
13	52	M	LGA	Multilobular	Unknown	Unknown	GRT	Temozolomide†	No	Focal unaware seizure	3	LEV 3,000	10	3			No	No
14	37	M	AA	Temporal	Unknown	Unknown	PR	Fotemustine‡	Yes†	Focal unaware seizure	2	VPA 1,000 LCM 400	10		4 (Side effects)	2	Aggressiveness	No
15	32	M	AA	Frontal	Mutated	Methylated	PR	Fotemustine§	Yes†	Focal aware seizure	30	LEV 3,000 LCM 400	6	20			No	Yes
16	45	F	AA	Multilobular	Not mutated	Methylated	PR	No	No	Focal unaware seizure	4	VPA 1,300 LCM 400	4	0.33			No	No
17	49	M	GBM	Occipital	Mutated	Unknown	GTR	Temozolomide‡	Yes†	Focal to bilateral tonic‐clonic	1	LEV 3,000	4	0			No	No
18	55	M	GBM	Parietal	Not mutated	Unknown	GTR	Other‡	No	Focal aware seizure	1	OXC 600 VPA 500 LCM 200 LEV 3,000	4		2 (Death)	1	No	Yes
19	56	F	GBM	Multilobular	Not mutated	Not methylated	PR	Temozolomide‡	Yes‡	Focal aware seizure	1	LEV 3,000	4	0			Vertigo (dose reduction)	Yes
20	49	M	GBM	Multilobular	Not mutated	Unknown	PR	Temozolomide‡	Yes†	Focal aware seizure	1	LEV 3,000	4	0.3			No	No
21	51	M	AA	Frontal	Not mutated	Not methylated	GTR	Temozolomide‡	Yes‡	Focal to bilateral tonic‐clonic	1	CBZ 400 LEV 3,000 CNZ 10	6	0.5			No	Yes
22	33	F	LGO	Multilobular	Mutated	Unknown	PR	Other‡	Yes†	Focal aware seizure	5	LEV 1,000	2		2 (Death)	0	No	Yes
23	36	F	MEN	Parietal	Unknown	Unknown	GTR	No	No	Focal aware seizure	2	ZNS 100	8	1			No	No
24	38	F	LGO	Parietal	Unknown	Unknown	PR	Temozolomide‡	Yes†	Focal aware seizure	15	CBZ 800 LCM 100	8	1			No	No
25	38	F	MET	Parietal	Unknown	Unknown	GTR	Other‡	Yes†	Focal unaware seizure	12	LEV 2,000 LCM 75 CNZ 10	4		2 (Side effects)	1	Vertigo/aggressiveness	Yes
26	46	F	MEN	Parietal	Unknown	Unknown	PR	No	Yes†	Focal to bilateral tonic‐clonic	8	LTG 400 VPA 1,000	8	5			No	No

Note

‐Histology = MEN: meningioma; LGG: low‐grade glioma; LGO: low‐grade oligodendroglioma; LGA: low‐grade astrocytoma; AA: anaplastic astrocytoma; OAO: oligoastrocytoma; HGG: high‐grade glioma; GBM: glioblastoma; MET: brain metastasis.

‐Surgery = PR: partial resection; GTR: gross total resection.

‐Chemotherapy = † before; ‡ before and during follow‐up; § during follow‐up; TMZ: temozolomide; CCNU: fotemustine; HDU: oncocarbide.

‐Radiotherapy = † before; ‡ before and during follow‐up; § during follow‐up.

‐AEDs (antiepileptic drugs): LEV: levetiracetam; VPA: valproic acid; OXC: oxcarbazepine; LTG: lamotrigine; TPM: topiramate; PB: phenobarbital; LCM: lacosamide; PER: perampanel.

During the follow‐up, 16 underwent chemotherapy and 11 radiotherapy; no other therapeutic modifications were made. Eleven had oncological disease progression evidenced using brain magnetic resonance (42.3%).

Five patients dropped out: 3 for disease progression and 2 for side effects: one for aggressiveness and one for vertigo and aggressiveness.

The mean daily PER dosage was 6.6 mg in the 21 patients who reached the final follow‐up and 6.4 mg in the ITT population.

Results on seizures frequency are reported for the ITT population (including all 26 treated patients) and for the 21 patients who reached 6 months of follow‐up and results are consistent.

The mean number of seizures/month in the 21 patients who reached the final follow‐up decreased from 10.8 ± 15.03 at baseline to 1.7 ± 4.34 (*p* = .01).

**FIGURE 1:**
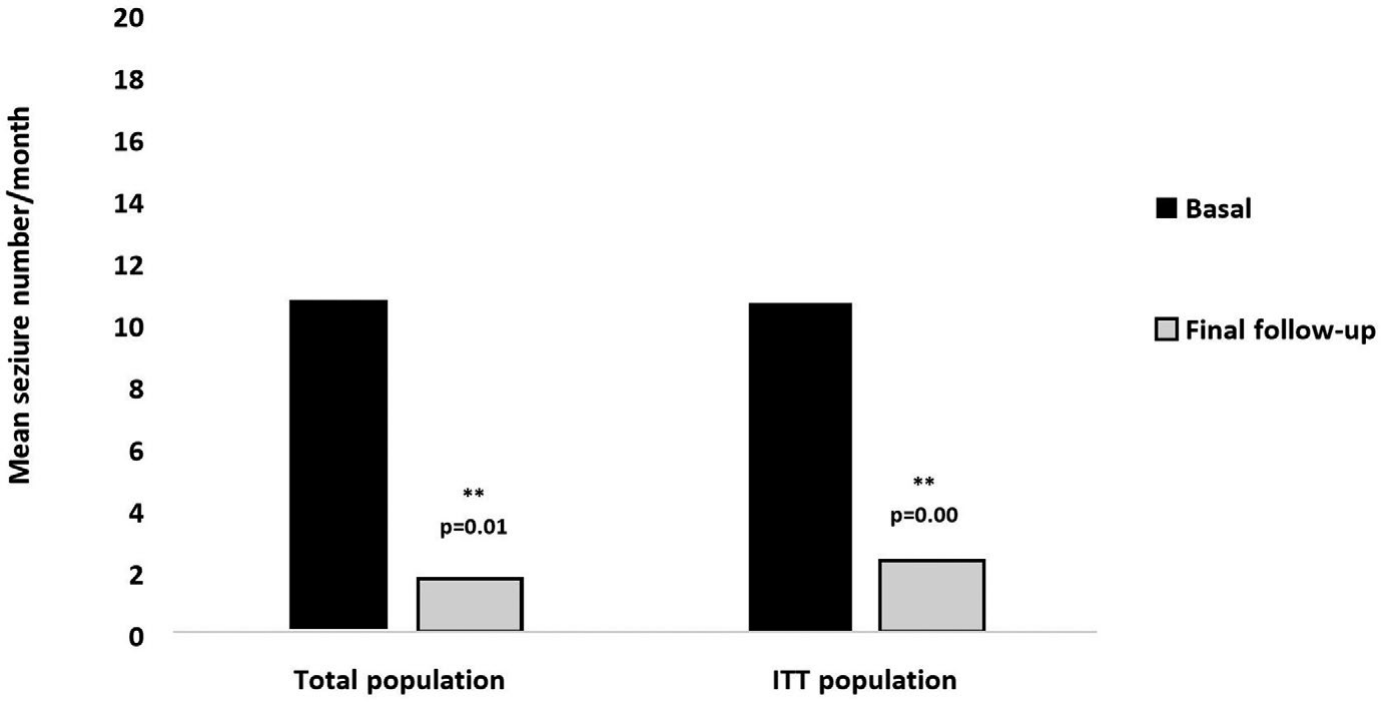
Comparison in mean seizure number/month between basal and final follow‐up evaluation in total population (*n* = 21) and in ITT population (*n* = 26)

In the ITT population (26 patients), the mean number of seizures reduced from 10.6 ± 14.27 at baseline to 2.3 ± 5.3 (*p* = .004) (last follow‐up available 2.4 months) (see Figure 1). Responder rate at 6 months was 95.2%: seven patients seizure‐free, 13 with a reduction ≥50%, and 1 remained stable (Table 1).

Four patients reported AEs (15.4%): 2 required PER dose reduction for vertigo (grade II of National Cancer Institute‐Common Terminology Criteria for Adverse Events‐NCI‐CTCAE) (Dunn‐Pirio et al., 2018), and 2 dropped out (1 due to aggressiveness/vertigo, and 1 due to vertigo).

The result at 6 months of neuropsychological, mood, and QoL questionnaires was not statistically different compared to baseline.

At baseline, quality of life questionnaires were administered in 20 patients because 2 patients had poor compliance and 4 aphasia. At the final follow‐up, 14 out of 20 were administered the quality of life questionnaire because 3 patients had disease progression and 3 dropped out (2 for side effect and 1 for poor compliance).

In these 14 patients, the comparison between baseline and final follow‐up did not show any statistically significant difference in QOLIE global score (basal: 61.2 ± 18.1; final follow‐up: 64.5 ± 20.7; *p* = .42), and values remained stable, within normal ranges (Table 2).

**TABLE 2:** Comparison between quality of life, neuropsychological, psychological tests and AEP profile before and after 6 months of treatment with PER in add‐on therapy

	Basal	6‐month follow‐up	*p*
	(Mean ± *SD*)	(Mean ± *SD*)	
Quality of life evaluation (*n* = 14) QOLIE 31‐P			
Seizure worry	45.1 ± 26.0	50.0 ± 27.5	.48
Quality of life	58.3 ± 16.0	62.6 ± 20.0	.38
Emotional well‐being	60.4 ± 16.3	61.2 ± 23.7	.86
Energy/fatigue	55.4 ± 20.3	55.7 ± 24.7	.96
Cognitive	68.0 ± 27.3	71.8 ± 24.5	.53
Meds effect	60.1 ± 28.5	60.8 ± 29.3	.93
Social functioning	67.2 ± 30.7	72.7 ± 30.3	.12
Global score	61.2 ± 18.1	64.5 ± 20.7	.42
Neurocognitive evaluation (*n* = 9) Neuropsychological tests			
MMSE	26.9 ± 2.5	27.0 ± 2.4	.34
Raven CPM	28.7 ± 7.1	30.1 ± 5.6	.20
Visual Search	48.4 ± 8.0	49.5 ± 4.4	.60
TMT A	48.4 ± 21.3	40.8 ± 14.7	.21
TMT B	97.8 ± 44.2	94.3 ± 33.7	.59
TMT B‐A	54.7 ± 29.6	55.6 ± 25.2	.75
Rey Auditory verbal learning test‐immediate recall	37.7 ± 15.4	38.7 ± 14.7	.54
Rey Auditory verbal learning test‐delayed recall	6.3 ± 4.4	6.6 ± 4.3	.35
Rey–Osterrieth Complex figure‐ copy	28.7 ± 7.9	28.2 ± 5.5	.83
Rey–Osterrieth Complex figure‐immediate recall	17.2 ± 9.5	17.4 ± 8.8	.84
Rey–Osterrieth Complex figure delayed recall	16.1 ± 9.0	16.9 ± 8.9	.15
Tower of London	30.0 ± 2.5	31.2 ± 2.9	.15
Phonemic fluency	29.3 ± 8.8	27.8 ± 8.1	.40
Categorial fluency	39.2 ± 12.0	39.3 ± 9.5	.93
Psychological state evaluation (*n* = 9) Symptom checklist‐90 (SCL‐90)			
SCL‐90 somatic	**0.70 ± 0.4**	**0.47 ± 0.4**	**.05**
SCL‐90 obsessive–compulsive	0.81 ± 0.7	0.58 ± 0.7	.26
SCL‐90 interpersonal sensibility	0.42 ± 0.4	0.30 ± 0.30	.42
SCL‐90 depression	0.72 ± 0.4	0.72 ± 0.8	.99
SCL‐90 anxiety	0.71 ± 0.6	0.58 ± 0.7	.35
SCL‐90 Hostility	0.66 ± 1.0	0.56 ± 0.8	.16
SCL‐90 phobia	0.32 ± 0.4	0.22 ± 0.2	.35
SCL‐90 Paranoic	0.27 ± 0.2	0.37 ± 0.5	.49
SCL‐90 psychoticism	0.41 ± 0.3	0.38 ± 0.4	.78
SCL‐90 sleep disturbances	0.81 ± 1.3	0.62 ± 0.9	.33
SCL‐90 global symptoms index	0.56 ± 0.4	0.46 ± 0.5	.44
AEDs' side effect evaluation (*n* = 9)			
AEP‐Adverse Event Profile	45.0 ± 12.4	41.7 ± 10.3	.14

Note

Coefficients set in bold indicate significant test differences between 6 months and baseline.

Abbreviations: MMSE, Mini‐Mental State Examination; Raven CPM, Raven colored progressive matrices; RAVLT, Rey Auditory Verbal Learning test; ROCF, Rey–Osterrieth complex figure; TMT B‐A, Trail Making Test Part B‐A; TMT A, Trail Making Test Part A; TMT B, Trail Making Test Part B; TOL—Tower of London.

At baseline, neuropsychological questionnaires were administered to 14 out of 26 patients, because 8 patients had poor compliance and 4 aphasia. At the final follow‐up, 9 out of 14 underwent a final evaluation, because 2 patients had disease progression, 2 dropped out for side effects, and 1 had poor compliance. In these 9 patients, the comparison between basaline and final follow‐up did not show any statistically significant difference, indicating performances stably included within normal ranges (Table 2).

At baseline, mood questionnaire (SCL‐90) and AEP‐adverse event profile were administered to 13 out of 26 patients, because 9 had poor compliance and 4 had aphasia. At the final follow‐up, 9 out of 13 patients underwent final evaluation because 1 had poor compliance, 2 dropped out due to side effects, and 1 had disease progression.

AEP mean scores did not show any statistically significant difference in the comparison between basal and final follow‐up evaluation (AEP basal: 45.0 ± 12.4, final follow‐up 41.7 ± 12.3, *p* = .14), indicating the presence of moderate AED induced side effects (Table 2).

SCL‐90 tests mean scores showed a statistically significant decrease in psychosomatic symptoms' scale (basal: 0.70 ± 0.4; final: 0.47 ± 0.4, *p* = .05) and no statistically significant difference in the other questionnaire domains between basaline and final follow‐up evaluation (SCL‐global score basal 0.5 ± 0.4; final follow‐up 0.4 ± 0.5, *p* = .44), indicating absence of any relevant psychopathological symptoms (Table 2).

In order to evaluate a potential correlation between seizure frequency during PER therapy and issues related to the oncological disease, we compared the decreasing number of seizures depending on: histology (low/high grade *p* = .73); surgical procedures (gross total resection/partial resection/biopsy *p* = .47); presence/absence of chemotherapy (*p* = .21), radiotherapy (*p* = .61) and progression disease (*p* = .65) during PER therapy; IDH1‐mutated/wild type (*p* = .77) and 06‐methylguanine‐DNA methyltransferase (MGMT) with or without promoter methylation (*p* = .95).

In all these, comparisons no significant differences were observed.

## 4 DISCUSSION

To date, there are very limited evidences on the efficacy of AEDs in the treatment of BTRE from randomized controlled trials (Perucca, 2013). Therefore, physicians are often driven for therapeutic choices by data obtained from other epilepsy subpopulations, or from noninterventional studies.

Among the newest AEDs, the antagonist activity of PER on AMPA glutamate receptor may constitute a basis for the use of this drug in the BTRE treatment.

We reported the results of an observational pilot study on 26 BTRE patients treated for 6 months with PER as add‐on therapy. We observed a reduction in seizure frequency in the 21 patients who reached the final follow‐up, from 10.8 ± 15.03 to 1.7 ± 4.34 (*p* = .01), and from 10.6 ± 14.27 to 2.3 ± 5.3 (*p* = .004) in the ITT population (26 patients) at the recommended dosage of PER treatment.

Responder rate at 6 months (21 patients) was 95.2, with 33.3% of patients who were seizure‐free after 6 months. Seizure frequency remained stable in 1 out of 21 patients, and none had seizures worsening.

Literature data on BTRE patient populations treated with PER as add‐on are very few; however, they indicated a good seizure response rate; Vecht and colleagues in a prospective study on 12 patients with low‐ and high‐grade gliomas and drug‐resistant epilepsy, assuming PER for 6 months for a median daily dose of 8 mg, reported an high seizure response rate in 9 out of 12 patients (75%), seizure freedom in 6 out of 12 patients (50%), improvement in cognitive functions and acceptable safety profile (Vecht et al., 2017). Izumoto and colleagues evaluated a case series of 12 patients with uncontrollable epilepsy related to both low‐ and high‐grade gliomas treated with PER as add‐on therapy and obtained 10 patients who achieved more than 50% seizure reduction and seizure freedom in 6 patients (60%) (Izumoto et al., 2018). Our results are in line with these evidences, indicating a good seizure response to PER treatment as add‐on in this patient population.

With reference to adverse events, literature data on non‐oncological epileptic patients and BTRE patients treated with PER report incidence of physical (Dunn‐Pirio et al., 2018; French et al., 2012, 2013; Izumoto et al., 2018; Krauss et al., 2012; Steinhoff et al., 2013) and behavioral disturbances (Coyle, Clough, Cooper, & Mohanraj, 2014; Ettinger et al., 2015; Fycompa, 2016; Rugg‐Gunn, 2014; Vecht et al., 2017). In order to monitor the possible onset of PER‐related side effects, we decided to use three types of measures: patients' subjective reports (classified according to National Cancer Institute‐Common Terminology Criteria for Adverse Events‐NCI‐CTCAE) (Cancer Therapy Evaluation Program, 2003), AEP‐Adverse event profile for physical domains (Gilliam et al., 2004) and SCL‐90 symptoms checklist for psychological state (Derogatis & Savitz, 2000). Regarding patients' subjective reports, 4 out of 26 patients (15.4%) referred presence of side effects. In two patients that reported vertigo (common AEs reported with PER) (Fycompa, 2017), PER was reduced, and in 2 patients that reported aggressiveness, PER was withdrawn. Concerning the evaluation of AEDs related physical side effects (AEP), we observed no statistically significant difference in AEP profile mean scores between basal and final follow‐up which values remain stable indicating the presence of moderate physical side effects (see Table 2). Regarding neuropsychiatric side effects (SCL‐90), we observed a significant decrease in psychosomatic symptoms' scale mean scores and stability in all domains explored by questionnaire, which values remain stable within normal values at basal and at final follow‐up, indicating low incidence of neuropsychiatric disturbances (see Table 2).

Studies on patients with BTRE treated with PER as add‐on indicate the presence of low to moderate side effect evaluated only by patients' subjective reports, which only in few cases required drug's withdrawal. Vecht et al. observed dizziness (33%) and drowsiness (16.6%) and withdrew PER only in 2 cases (16.6%) (Vecht et al., 2017), Izumoto et al. observed 2 patients with dizziness (16.6%) and withdrew PER in only 1 case (8.3%) (Izumoto et al., 2018), Dunn‐Pirio et al. described the appearance of several side effects such as fatigue (63%) and dizziness (25%) during their fast titration period on all 8 patients enrolled but only one patient required PER dose reduction (Dunn‐Pirio et al., 2018). Our results, obtained not only through patients' subjective reports but also through a self‐report multi‐item questionnaire (AEP), are in line with literature evidences indicating low incidence of side effect of PER as add‐on in BTRE patients already in polytherapy with other AEDs.

As for the possibility that PER efficacy remains stable during oncological disease progression, our results indicate that despite the high number of patients with progression disease during the follow‐up (11 patients, 42.3%), the efficacy of PER on seizure control remains high, also in the group of patients with progression disease (Table 1).

Furthermore, our results did not evidence that the efficacy of PER on seizure control could be influenced by factors related to brain tumors, such as systemic therapy, oncological progression, different histology and malignancy, surgical procedures. This results should be cautiously considered because of low power due to small sample size and short follow‐up (only 6 months).

Regarding the molecular indices analysis, in the different two groups (IDH1‐mutated/wild type, MGMT with or without promoter methylation) we did not observe significative differences. Our results differ from the study results of Dunn‐Pirio et al. (2018) in which they found that, between patients with a decrease in seizure activity, the majority had IDH1‐mutant tumors. This difference could be caused by our low patient number, because for just 15 out of 26 patients we had the analysis for IDH1 and for 11 patients for MGMT.

Regarding QoL evaluation, we did not observe statistically significant differences in QOLIE 31 P mean scores, which values remain within normal ranges at basal and at final follow‐up evaluations (Table 2).

Literature data on BTRE patients indicate a correlation between good seizure control and improved scores in Quality of life questionnaire (Maschio & Dinapoli, 2012). Probably, in our sample we did not observe significant improvements in questionnaire mean scores because 9 out of 14 patients also performed concomitant oncological treatments (6 out of 14 CT; 2 out of 14 RT) and 2 other patients were in disease progression. However, the good seizure control obtained by patients with PER in add‐on at final follow‐up, contributed to keep QOLIE 31 mean values stable despite the influence of the abovementioned variables, indicating that PER had no impact on perceived quality of life in our patients population.

Regarding cognitive performances, we observed stability in patients' mean scores and at final follow‐up compared to baseline. This result is in line with the only one study in literature which shows a low impact of PER on cognitive functions in glioma patients, tested with a short computerized battery (Vecht et al., 2017).

However, our data on tests and questionnaires cannot be generalized due to the small number of patients who repeated control tests at 6 months. With reference to this specific aspect, it could be useful to administer a brief neuropsychological test battery or brief and specific questionnaires in order to avoid the high number of patients with poor compliance, as suggested by literature data (Newton & Maschio, 2015).

## 5 CONCLUSION

In our study, we observed good efficacy on seizure control without negative effects on cognition and on QoL of PER in patients with BTRE.

Despite the limitations due to the small number of patients, PER could be a therapeutic option in BTRE patients due to responders' high rate and number of seizure‐free patients.

These results need further studies with a longer follow‐up to confirm this high responder rate and the possible correlation with molecular indices, which could be a starting point for truly tailored therapies for patients with BTRE.

## CONFLICT OF INTEREST

Dr. Marta Maschio has received support for travel to congresses from EISAI srl; has participated in scientific advisory boards for EISAI; has participated in pharmaceutical industry‐sponsored symposia for UCB Pharma; and has received research grants from UCB Pharma. No conflicts of interest are declared for the other Authors.

## AUTHORS' CONTRIBUTIONS

MM: study design; collection of the cases; writing of the manuscript; editing of the manuscript; AZ: collection of the cases, writing of the manuscript; AM: collection of the cases, writing of the manuscript; DG: study design, statistical analysis; TK: collection of the cases; EG: collection of the cases; VV: collection of the cases; SZ: collection of the cases.

## Data Availability

At the Biostatistic Unit of Regina Elena National Cancer Institute all dataset analyzed for the current study is available.
